# Assessment of acute and non-acute suicide crisis symptoms: Validation of the Korean version of the acute suicidal affective disturbance inventory

**DOI:** 10.3389/fpsyg.2022.1034130

**Published:** 2022-11-18

**Authors:** Yuna Oh, Sungwoo Lee, Megan L. Rogers, Sungeun You

**Affiliations:** ^1^Department of Psychology, Chungbuk National University, Cheongju, Chungbuk, South Korea; ^2^Department of Psychology, Texas State University, San Marcos, TX, United States

**Keywords:** acute suicidal crisis, acute suicidal affective disturbance, acute suicidal affective disturbance inventory, reliability, validity

## Abstract

Suicide risk assessment is predominantly based on assessing current/recent suicidal ideation and past suicidal behavior. However, suicidal ideation and lifetime suicide attempt are poor predictors of imminent suicide risk or crisis. The acute suicidal affective disturbance inventory-lifetime (ASADI-L) was developed to assess symptoms of acute suicidal affective disturbance, which includes a drastic increase in suicidal intent, perceptions of social and/or self-alienation, hopelessness, and overarousal. However, the ASADI-L has not yet been validated in a Korean population. Also, the ASADI-L has only been validated for people who experience a drastic increase in suicidal intention over the course of hours or days (i.e., the acute suicidal intention group) and not validated for those who experience suicidal intention for a longer period (i.e., the non-acute suicidal intention group). Thus, the aims of this study were to (1) validate the ASADI-L in a sample of Korean community adults; and (2) compare clinical characteristics of the acute and non-acute suicidal intention groups. Among 1,675 community adults, data from 682 participants who reported a lifetime drastic increase in suicidal intent were analyzed. Results indicated that the ASADI-L has relevant reliability, validity, and a unidimensional factor structure. The acute suicidal intention group had higher ASAD symptoms as well as clinical symptoms than the non-acute group, but the two groups did not differ in history of suicide attempt. Overall, these findings suggest that the ASADI-L is a valid measure of acute and non-acute suicidal affective disturbance among Korean adults. Further investigation of the differences in acute and non-acute suicide risk is warranted.

## Introduction

Assessments of suicidal crises have traditionally focused on current suicidal thoughts and past suicidal behavior. However, only some of those who think of suicide actually engage in suicidal behavior (Nock et al., 2008), and accumulating studies show that it is difficult to predict suicidal behavior using current suicidal thoughts and past suicidal behavior as well as well-known risk factors such as depression due to the low base rate of suicide (Silverman and Berman, 2014; Franklin et al., 2017). Thus, there has been an emerging demand for classification criteria on the phenomenology and etiology of short-term suicide risk as an independent mental condition to properly assess and intervene in acute suicidal crises (Oquendo and Baca-Garcia, 2014; Rogers et al., 2017a; Fehling and Selby, 2021). Joiner et al. (2018) suggested potential advantages to establishing specific diagnostic criteria for suicidality. First, the diagnostic criteria could be utilized to assess and intervene upon suicidal crises by specifying their clinical characteristics and precipitating factors. Second, a suicide-specific diagnosis would contribute to enhancing personal safety and preventing suicide by enabling appropriate clinical intervention for those at risk of suicide. Finally, in the case of hospitalization due to suicidal behavior, a thorough safety plan after discharge can be established to reduce the legal responsibility of clinicians.

The Diagnostic and Statistical Manual of Mental Disorders 5th Edition (DSM-5; American Psychiatric Association, 2013) included “suicidal behavior disorder (SBD)” in the section of the “conditions for further study” and defined SBD as “whether the individual has made a suicide attempt within the last 2 years.” In the updated version, the DSM-5 Text Revision (DSM-5-TR; American Psychiatric Association, 2022), a diagnostic code was assigned to suicidal behavior and nonsuicidal self-injury, and it was placed in the “other conditions that may be a focus of clinical attention” chapter. However, this DSM-5 diagnostic criterion for SBD has been criticized for several reasons. Ribeiro et al. (2016) noted that SBD predicts future suicidal behavior using past suicide attempts alone, showing low predictive validity. Moreover, SBD has been criticized for failing to measure acuity (e.g., sudden suicidal crises) as well as reflect behaviors during acute suicidal crises (Tucker et al., 2016; Rogers et al., 2019a).

In this context, diagnostic criteria for acute suicide crisis were proposed by two independent suicide research groups. One is “Acute Suicidal Affective Disturbance” (ASAD; Tucker et al., 2016), the focus of this study, and the other is “Suicide Crisis Syndrome” (SCS; Galynker et al., 2017). Both ASAD and SCS diagnostic criteria include clinical symptom clusters people experience at the time of acute suicide crisis, whereas SCS differs from ASAD in that it does not inquire about overt suicidal ideation or intention (Rogers et al., 2017b, 2019a,b). Joiner and colleagues proposed diagnostic criteria for ASAD (Stanley et al., 2016; Tucker et al., 2016; Rogers et al., 2017b, 2019a). ASAD was proposed to encompass four major diagnostic criteria for assessing time-limited acute suicidal crises: (1) a drastic increase in suicidal intent over the course of hours or days (as opposed to weeks or months); (2) one (or both) of the following: marked social alienation (e.g., social withdrawal, disgust with others, perceptions that one is a liability on others) and/or self-alienation (e.g., self-hatred, perceptions that one’s psychological pain is a burden); (3) perceptions that one’s suicidality, social alienation, and self-alienation are hopelessly unchangeable; and (4) two (or more) manifestations of overarousal (i.e., agitation, irritability, insomnia, nightmares).

Based on the ideation-to-action framework of Klonsky and May (2014) and Tucker et al. (2016) noted that those who have suicidal thoughts lead to suicidal behavior with a drastic increase in suicidal intention over the course of hours or days. Second, social/self-alienation were included in ASAD symptoms according to the interpersonal theory (Joiner, 2005; Van Orden et al., 2010) that social withdrawal increases suicidal desire, and the studies that self-alienation can be a major symptom of suicidal crisis (Chu et al., 2017). The third symptom of ASAD is hopelessness, which involves the perception that one’s drastically increasing suicidal intention, social alienation, and self-alienation are unlikely to disappear or are hopelessly unchangeable. Hopelessness has been regarded as an important suicidal crisis symptom in Beck’s cognitive model for suicide (Wenzel and Beck, 2008; Wenzel et al., 2009). Finally, overarousal was included as a symptom of ASAD given evidence that agitation, irritability, and sleep disturbances are important components of suicide risk (Chu et al., 2015).

To measure symptoms of ASAD, Tucker et al. (2016) developed the acute suicidal affective disturbance inventory-lifetime (ASADI-L). Tucker et al. reported that the ASADI-L consists of one factor in a group of college students in the US. Other studies also have shown that ASAD symptoms form a one-factor structure for psychiatric outpatients and inpatients (Stanley et al., 2016; Rogers et al., 2017a). Stanley et al. (2016) and Rogers et al. (2017a) re-verified the one-factor structure and validity of ASAD symptoms with 1,442 psychiatric outpatients and with 343 psychiatric outpatients and 7,698 inpatients, respectively. Moreover, various precipitating factors of ASAD have been identified and tested, including negative cognitive styles (Rogers et al., 2019c) and suicide-specific rumination (Rogers and Joiner, 2018).

Tucker et al. (2016) requested the participants who had reported they experienced a drastic increase in suicidal intent “over the course of hours or days,” among those who reported ever having such an experience, to respond to subsequent questions regarding social/self-alienation, hopelessness, and overarousal on the ASADI-L. In this respect, the same study defined an experience of suicidal intention within a short period as a suicidal crisis. However, when this method of measure is applied, the opportunity to intervene in the other high-risk group, where participants experience a drastic increase in suicidal intention long-term, may be missed. Thus, the ASADI-L needs to be validated for people who experience suicidal intention for a longer period (i.e., the non-acute suicidal intention group) in addition to those who experience a drastic increase in suicidal intention over the course of hours or days (i.e., the acute suicidal intention group).

To build on previous research and to extend the construct of ASAD to a Korean population, for whom suicide risk is elevated, the purpose of this study aimed to test the validity of the Korean translation of the ASADI-L and examine its reliability and validity in the Korean community population. Based on previous studies (Stanley et al., 2016; Tucker et al., 2016; Rogers et al., 2017a), we hypothesized that the Korean version of the ASADI-L would have a one-factor structure and strong internal consistency, convergent/discriminant validity, and criterion validity. Furthermore, this study explored, with no *a priori* hypotheses, whether there were any differences in ASAD symptom severity or clinical characteristics between participants with acute and non-acute suicidal intention.

## Materials and methods

### Participants

Using an online Qualtrics survey, the participants were recruited through various social media sites (e.g., Facebook, Instagram, local or national community sites, university websites etc.) from January to June 2021. This study is part of a longitudinal study to identify predictive factors for suicide crisis in a Korean community sample. The study procedure was approved by Chungbuk National University Institutional Review Board (CBNU202010-HR-0164). A total of 1,675 community adults completed the survey and among them, 1,357 (81.01%) endorsed them as women. Out of 1,675, data from 682 participants who responded “Yes” to ASADI-L Question 1 (“In your lifetime, have you ever experienced any thoughts of suicide?”) and Question 2a (“In your lifetime, have you ever experienced a drastic increase in your intent to kill yourself?”) were analyzed. Average age of participants was 30.0 years (*SD* = 6.5, ages 19–61). Approximately 90% of participants were in their 20 s and 30 s: 328 people were in their 20 s (48.1%), including a 19-year-old, and 281 people (41.2%) were in their 30 s. Moreover, there were 605 women (88.7%), comprising the majority. Among 1,675 participants, those who did not include in this study due to no endorsement of suicidal ideation were significantly older, *t* = 5.42, *p* < 0.001, and comprised of less women, *χ*^2^ (2, *N* = 1,675) = 45.40, *p* < 0.001, than those who included.

### Instruments

#### ASADI-L

The ASADI-L is a measure developed by Tucker et al. (2016) to assess ASAD symptoms at their worst point across one’s lifetime. The ASADI-L measures the symptoms of drastically increasing suicidal intent, social/self-alienation, hopelessness, and overarousal. Although the ASADI-L in Tucker et al. consists of a total of 28 questions, this study used a revised version of the ASADI-L having 24 questions, excluding Questions regarding “planning and preparing for suicide attempt,” through email communication with the original measure developer (Joiner, personal communication, September 2019).

In this study, those who reported the experience of a drastic increase in suicidal intention (i.e., those who responded yes to ASADI-L item 2a) were asked to respond to the entire ASADI-L questionnaire. Participants who reported the experience of a drastic increase in suicidal intention “over the course of hours or days” were referred to as the group with acute suicidal intention, whereas those with longer durations were referred to as the group with non-acute suicidal intention (i.e., those who responded yes ASADI-L item 2a and no to ASADI-L item 2b, respectively).

ASAD symptom severity is calculated using the formula: ASADI-L item 2c*10 + (4b*4c + 5b*5c + 6b*6c)/3 + 7b*7c + [8 + 9 + 10 + 11a]/4*10 (Table 1 for item information), where a higher total score indicates higher ASAD symptom severity (Rogers et al., 2019c). The four domains of suicidal intent (2c), social alienation (4b–6c), self-alienation (7b–7c), and overarousal (8–11a), each calculated with a score of 0–100, are summed, resulting in a total ASAD symptom severity score ranging from 0 to 400. Tucker et al. (2016) presented that the internal consistency coefficient (Cronbach’s α) of the ASADI-L was determined to be as high as 0.97. In this study, it was determined to be 0.88. The Korean version of the ASADI-L measure used in this study is provided in the Supplementary material.

**Table 1:** Descriptive statistics of the acute suicidal affective disturbance inventory-lifetime (ASADI-L).

		Total *N* = 682		Acute *n* = 262		Non-acute *n* = 420		Statistics	*p*	Cohen’s *d*
		*M*	*SD*	*M*	*SD*	*M*	*SD*			
2c	Drastic increase in suicide intent (highest intent)	6.72	2.02	6.95	2.04	6.58	2.01	2.35	0.019	0.185
4b	Severity of social disconnection	5.94	3.44	6.33	3.33	5.70	3.49	2.36	0.019	0.186
4c	Immutability of social disconnection	5.60	3.51	5.88	3.41	5.43	3.56	1.65	0.099	0.130
5b	Severity of disgust toward others	3.93	3.97	4.63	3.96	3.50	3.92	3.64	<0.001	0.287
5c	Immutability of disgust toward others	3.72	3.94	4.33	3.95	3.33	3.89	3.24	<0.001	0.255
6b	Perceived burdensomeness	5.10	3.91	5.62	3.90	4.77	3.88	2.80	0.005	0.220
6c	Immutability of perceived burdensomeness	4.76	3.80	5.14	3.81	4.53	3.78	2.05	0.041	0.161
7b	Disgust toward self	5.52	3.88	5.95	3.76	5.25	3.94	2.32	0.021	0.182
7c	Immutability of disgust toward self	5.12	3.81	5.50	3.74	4.88	3.84	2.09	0.037	0.125
8	Severity of agitation	6.76	2.48	6.95	2.46	6.64	2.49	1.59	0.113	0.129
9	Severity of irritation	7.33	2.56	7.53	2.46	7.20	2.61	1.63	0.103	0.153
10	Severity of insomnia	6.74	2.92	7.01	2.85	6.56	2.96	1.95	0.052	0.222
11a	Severity of nightmares	5.08	3.40	5.54	3.31	4.79	3.43	2.82	0.005	0.263
	ASAD total score	210.32	81.08	223.35	83.02	202.20	78.86	3.34	<0.001	0.125

ASAD total score was calculated by the formula: 2c*10 + (4b*4c + 5b*5c + 6b*6c)/3 + 7b*7c + [8 + 9 + 10 + 11a]/4*10. In this formula, 2c highest intent was implemented. Acute (2a = yes, 2b = yes) and non-acute (2a = yes, 2b = no) suicidal intention groups were divided based on responses to the question 2a (in your lifetime, have you ever experienced a drastic increase in your intent to kill yourself?) and 2b (if yes, did this drastic increase in intent to kill yourself occur over the course of hours or days, not over the course of weeks or months?).

#### Columbia suicide severity rating scale - screen version

The C-SSRS is a self-report measure developed to assess the severity of suicide risk (Posner et al., 2008). This study used the C-SSRS to determine the convergent validity of the ASADI-L along with lifetime history of suicide attempts. We added questions on history of suicide attempts and number of lifetime suicide attempts in addition to the C-SSRS screen version. This measure consists of five questions measuring the severity of suicidal ideation over the past month and during the respondent’s lifetime and one question measuring suicide attempt preparation behavior over the past 3 months and during the respondent’s lifetime. “Yes/No” questions are utilized for each question, and the severity of suicidal ideation is measured by the maximum value of the score assigned to each question (with a possible range of 0 to 5). Cases with suicidal ideation severity scores of 4 or higher are classified in the high-risk group for suicide (Posner et al., 2008). Internal consistency (α) of the C-SSRS was 0.93 in the study of Posner et al. (2011) and 0.75 in this study.

#### Patient health questionnaire-9

The PHQ-9 is a 9-item self-report measure of the severity of depressive symptoms over the past 2 weeks (Spitzer et al., 1999). This study utilized the Korean version of the measure validated by An et al. (2013) to check the discriminant validity of the ASADI-L. The internal consistency (α) of the Korean version of the PHQ-9 was determined to be 0.95 in the study of An et al. and 0.91 in this study.

#### Generalized anxiety disorder 7-item scale

The GAD-7 is a 7-item self-report measure of the severity of anxiety symptoms over the past 2 weeks (Spitzer et al., 2006). This study utilized the Korean version of the measure validated by Ahn et al. (2019) to check the discriminant validity of the ASADI-L. The internal consistency (α) of the Korean version of the GAD-7 was determined to be 0.92 in the study of Ahn et al. (2019) and 0.91 in this study.

#### Inventory of statements about self-injury

The ISAS is a self-report measure developed by Klonsky and Glenn (2009) to assess non-suicidal self-injury. This study utilized the Korean version of the ISAS scale validated by Chu and Lee (2018) to check the discriminant validity of the ASADI-L. The ISAS scale consists of three main parts: (1) the frequency of self-injurious behavior; (2) the functions of self-injury, composed of social and intrapersonal functions; and (3) two open-ended questions that require participants to additionally describe the self-injurious behavior, providing more detailed information than the previous questions. The internal consistency coefficients for the functions of self-injury in the original measure were 0.88 for social function and 0.80 for intrapersonal function, whereas those in the Korean version of the self-injury measure were determined to be 0.77 for social function and 0.77 for intrapersonal function. This study utilized only the frequency question of self-injurious behavior, excluding items 6 (behavior not beneficial to wound healing) and 11 (hair pulling) according to the criteria of Hooley et al. (2020).

### Data analytic strategy

All statistical analyses were performed using Jamovi version 2.3.2. Descriptive statistics were first computed to examine the demographic and clinical characteristics of participants, both for the overall sample and stratified by those in the acute and non-acute suicidal intent groups. Then, a series of independent samples *t*-test was conducted to determine the differences between the acute and non-acute suicidal intention groups in terms of demographic and clinical characteristics, as well as each ASAD symptom.

Next, a confirmatory factor analysis (CFA) was performed to confirm the goodness of fit of the proposed unidimensional factor structure of the ASADI-L. The comparative fit index (CFI) and Tucker Lewis index (TLI) shows good fit at 0.90 or higher (Hair et al., 1998), and the standardized root mean square residual (SRMR) reflects good fit at 0.08 or lower (Hu and Bentler, 1999). The goodness of fit for the root mean square error of approximation (RMSEA) is excellent at 0.05 or lower, suitable at 0.08 or lower, and low at 0.10 or higher (Hair et al., 1998). The model was modified after setting the modification indices to a criterion of 10 or higher according to the evidence that a correlation between error terms is allowed if the content of the questions is similar in the CFA (Brown and Moore, 2012). The specific modifications were conducted as follows: (1) to measure social/self-alienation, ASADI-L items on alienation in interpersonal relationships (4b–4c), disgust in interpersonal relationships (5b–5c), perceptions that one is a liability on others (6b–6c), and self-hatred (7b–7c) were linked; (2) ASADI-L item 4c–7c all measure the level of hopelessness regarding social/self-alienation and thus the correlations between the errors of all c items were linked (4c–5c, 4c–6c, 4c–7c, 5c–6c, 5c–7c, 6c–7c); (3) the items measuring sleep (10–11) were connected. To test the validity of the ASADI-L, a Pearson correlation analysis with the suicide-related mental health scale was conducted. The correlation was interpreted as being low at 0.10 or lower, moderate between 0.20 and 0.40, and high at 0.50 or higher (Cohen, 1988). Finally, the internal consistency coefficient (Cronbach’s *α*) was calculated to verify the reliability of the measure.

Moreover, a one-way analysis of variance (ANOVA) was performed to determine the difference in the severity of ASAD symptoms between the suicide attempt groups. Specifically, the difference in ASAD symptoms between the groups was examined by dividing the groups into no attempt, single attempt, and multiple (two or more) suicide attempts.

## Results

### Descriptive and clinical characteristics of the acute and non-acute suicidal intent groups

Table 2 presents demographic and clinical characteristics of all participants. As shown in Table 2, 24.5% (*n* = 167) of 682 study participants reported a history of suicide attempts during their lifetime, and 39.3% (*n* = 268) had a score of 4 or higher on the C-SSRS suicidal ideation severity scale, corresponding to the high-risk group. Although the average age of the acute suicidal intention group was higher than that of the non-acute suicidal intention group, *t* (687) = 12.06, *p* = 0.034, there was no significant difference in the gender distribution, *χ*^2^ (2, *N* = 682) = 5.41, *p* = 0.067, between the two groups. The acute suicidal intention group showed significantly higher levels than those of the non-acute suicidal intention group in the ASADI-L, *t* (680) = 3.34, *p* < 0.001; C-SSRS suicidal ideation severity, *t* (520) = 2.92, *p* = 0.004; depression, *t* (512) = 5.99, *p* < 0.001; anxiety, *t* (674) = 5.29, *p* < 0.001; and frequency of non-suicidal self-injury, *t* (407) = 3.52, *p* < 0.001. However, there was no significant difference in history of suicide attempts between the acute and non-acute suicidal intention groups, *χ*^2^ (1, *N* = 681) = 2.72, *p* = 0.099.

**Table 2:** Demographic and clinical characteristics of the acute and non-acute suicidal intention groups.

	Total *N* = 682		Acute *n* = 262		Non-acute *n* = 420		Statistics	*p*	Cohen’s *d*
	*M*	*SD*	*M*	*SD*	*M*	*SD*			
Age	30.03	6.46	30.75	7.44	29.58	5.72	12.06	0.034	
Gender (*n*, %)							5.41	0.067	
Women	605	88.71	225	85.88	380	90.48			
Men	75	11.00	37	14.12	38	9.05			
Others	2	0.29	0	0	2	0.48			
ASADI-L^a^	210.32	81.07	223	83.02	202.20	78.85	3.34	<0.001	0.263
C-SSRS^b^	3.22	1.52	3.45	1.58	3.09	1.46	2.92	0.004	0.232
C-SSRS^b^ ≥4 (*n*, %)	268	39.30	126	48.09	142	33.81	13.80	<0.001	
PHQ-9^c^	10.90	6.93	12.9	7.13	9.66	6.51	5.99	<0.001	0.478
GAD-7^d^	8.32	5.79	9.78	5.87	7.41	5.56	5.29	<0.001	0.418
ISAS^e^	71.35	120.00	93.6	146.03	57.54	98.29	3.52	<0.001	0.289
Suicide attempt (*n*, %)	167	24.49	73	27.86	94	22.38	2.72	0.099	

Acute (2a = yes, 2b = yes) and non-acute (2a = yes, 2b = no) groups were divided based on responses to the questions 2a (In your lifetime, have you ever experienced a drastic increase in your intent to kill yourself?) and 2b (if yes, did this drastic increase in intent to kill yourself occur over the course of hours or days, not over the course of weeks or months?); ^a^ASADI-L, acute suicidal affective disturbance inventory-lifetime; ^b^Columbia-suicide severity rating scale (C-SSRS), lifetime suicide ideation severity; ^c^patient health questionnaire-9 (PHQ-9); ^d^generalized anxiety disorder 7-item scale (GAD-7); ^e^inventory of statements about self-injury (ISAS); *t*-statistics were used to compare the means of two groups and Chi-square statistics were used to compare categorical variables.

Table 1 presents the mean and SD for each ASADI-L item. The acute suicidal intention group showed significantly higher scores than the non-acute suicidal intention group on most ASADI-L items (including a drastic increase in suicidal intent; the severity of social disconnection and nightmares; and the severity and perceived intractability of disgust with others, perceived burdensomeness, and self-disgust), as well as significantly higher total ASAD symptoms. However, there was no significant difference between the groups in the average scores regarding the immutability of social disconnection, the severity of agitation, irritability, and insomnia.

### Factor structure of the ASADI-L: CFA

To verify the goodness of fit for the factor structure of the ASADI-L, a CFA was conducted (Table 3). According to the analysis results, the adjusted goodness-of-fit indices of the one-factor model of the ASADI-L for the entire sample were determined as follows: *χ*^2^ (54, *N* = 682) = 138.45, *p* < 0.001, CFI = 0.986, TLI = 0.980, SRMR = 0.037, and RMSEA = 0.047, 90% *CI* (0.038, 0.058), indicating good model fit. The adjusted goodness-of-fit indices for the acute suicidal intention group were determined as follows: *χ*^2^ (57, *n* = 262) = 117.56, *p* < 0.001, CFI = 0.975, TLI = 0.966, SRMR = 0.045, RMSEA = 0.064, 90% *CI* (0.047, 0.080), showing a suitable goodness of fit, whereas those of the non-acute suicidal intention group were determined as follows: *χ*^2^ (54, *n* = 420) = 100.58, *p* < 0.001, CFI = 0.988, TLI = 0.982, SRMR = 0.045, RMSEA = 0.045, 90% *CI* (0.031, 0.059), indicating a good model fit for each subgroup as well as for the full sample.

**Table 3:** Confirmatory factor analysis of the one-factor model of the ASADI-L.

	*N*	*χ*2	*df*	CFI	TLI	SRMR	RMSEA	RMSEA 90% CI	
								Lower	Upper
Total	682	138.45	54	0.986	0.980	0.037	0.047	0.038	0.058
Acute	262	117.56	57	0.975	0.966	0.045	0.064	0.047	0.080
Non-acute	420	100.58	54	0.988	0.982	0.039	0.045	0.031	0.059

Acute (2a = yes, 2b = yes) and non-acute (2a = yes, 2b = no) suicidal intention groups were divided based on responses to the question 2a (in your lifetime, have you ever experienced a drastic increase in your intent to kill yourself?) and 2b (if yes, did this drastic increase in intent to kill yourself occur over the course of hours or days, not over the course of weeks or months?); CFI, comparative fit index; TLI, Tucker Lewis index; SRMR, standardized root mean square residual, RMSEA, root mean square error of approximation.

### Validity of the ASADI-L

To test the convergent and discriminant validity of the ASADI-L, correlations were computed between the ASADI-L and other measures of suicidal ideation and psychopathology (Table 4). The ASADI-L was positively and moderately correlated with C-SSRS lifetime suicidal ideation severity (*r* = 0.41, *p* < 0.001), depression symptoms (*r* = 0.36, *p* < 0.001), anxiety symptoms (*r* = 0.36, *p* < 0.001), and non-suicidal self-injury (*r* = 0.34, *p* < 0.001), supporting both the convergent and discriminant validity of ASAD from other forms of psychopathology.

**Table 4:** Correlations among the ASADI-L and suicide-related measures.

	1	2	3	4	5
1. ASADI-L	-				
2. C-SSRS	0.41***	-			
3. PHQ-9	0.36***	0.29***	-		
4. GAD-7	0.36***	0.26***	0.79***	-	
5. ISAS	0.34***	0.24***	0.32***	0.33***	-
*M*	210.32	3.22	10.90	8.32	71.35
*SD*	81.07	1.52	6.93	5.79	120.00

ASADI-L, acute suicidal affective disturbance inventory-lifetime; C-SSRS, Columbia-suicide severity rating scale, lifetime suicide ideation severity; PHQ-9, patient health questionnaire-9; GAD-7, generalized anxiety disorder 7-item scale; ISAS, inventory of statements about self-injury. ***p < 0.001.

Additionally, a one-way ANOVA was conducted to determine whether the ASADI-L differentiated participants with no past suicide attempts, those with a history of a single attempt, and those with a history of multiple attempts (Table 5). There was a significant difference in the severity of ASAD symptoms among the three groups, *F* (2, 678) = 43.0, *p* < 0.001. Specifically, the severity of ASAD symptoms was higher in the groups with single (*M* = 246.83, SD = 76.20) or multiple (*M* = 265.54, *SD* = 76.38) past suicide attempts than the group with no history of suicide attempts (*M* = 195.04, *SD* = 76.63, *p*s < 0.001). However, there was no difference in the severity of ASAD symptoms between the single attempt and multiple attempt groups (*p* = 0.261).

**Table 5:** Comparisons of the ASADI-L among people with no suicide attempt, single suicide attempt and multiple suicide attempt.

Suicide attempt	ASADI-L		Statistics	*η* ^2^	*Post-hoc* Tukey
	*M*	*SD*			
None^a^	195.04	76.63	*F* (2, 678) = 43.00***	0.11	a < b, a < c
1^b^	246.83	76.20			
2 or more^c^	265.54	76.38			

ASADI-L, acute suicidal affective disturbance inventory-lifetime; ^a^No history of suicide attempt group; ^b^Single attempter group; ^c^Multiple attempter group. ***p < 0.001.

### Reliability of the ASADI-L

To verify the reliability of the ASADI-L, the internal consistency coefficient (Cronbach’s *α*) was checked for the total sample (*N* = 682), the acute suicidal intention group (*n* = 262), and the non-acute suicidal intention group (*n* = 420). Internal consistency (*α*) was good to high in all groups: 0.88 in the total sample, 0.90 in the acute suicidal intention group, and 0.87 in the non-acute suicidal intention group.

## Discussion

The DSM-5 describes the presence of suicide attempts within the past 2 years as a diagnostic criterion for SBD (American Psychiatric Association, 2013). However, measuring suicidal risk only with past suicidal behavior has limited predictive ability, and it cannot measure sudden or imminent suicidal crises. Tucker et al. (2016) developed the ASADI-L to assess acute suicidal crises, which include symptoms of social/self-alienation, hopelessness, and overarousal, along with a drastic increase in suicidal intention over the course of hours or days. We extended work on the ASADI-L by developing and validating the Korean version of the ASADI-L in a Korean community adult population. Results of the present study indicated that the Korean version of the ASADI-L is unidimensional, consistent with the results of Tucker et al. The factor structure of the ASADI-L was comparable among participants with a history of drastic and rapid increases in suicidal intent over the course of hours or days (acute suicidal intention group) and those with drastic and rapid increases in suicidal intent for a longer period (non-acute suicidal intention group). Both groups showed comparable validity and reliability. This suggests that the Korean version of the ASADI-L is a valid measure of affective disturbance during suicidal crisis regardless of the duration of a drastic increase in suicidal intention.

The correlations of ASAD symptoms with C-SSRS suicidal ideation severity and self-injurious behavior were 0.40 and 0.35, respectively. The correlation of ASAD symptoms with depression and anxiety symptoms was approximately 0.35, which is similar to the results of previous studies (Tucker et al., 2016; Rogers et al., 2017a, 2019b; Buckner et al., 2020). The moderate correlation between ASAD symptoms and depression/anxiety can be explained in that there are distinct but overlapping symptoms such as sleep problems and agitation. This is also consistent with the result from a network analysis that ASAD symptoms are distinct from depression and anxiety (Rogers et al., 2019b). The result that ASAD symptoms have moderate correlations with suicidal ideation measured by the conventional measure of suicide risk suggests that the two measurements assess related but different concepts, supporting discriminant validity of the ASADI-L. The ASADI-L also had only moderate correlations with suicide-related measures, supporting its discriminant validity.

Furthermore, our results indicated that, among those who have experienced a drastic increase in suicidal intention, approximately one fourth reported a history of suicide attempt. ASAD symptoms were significantly high in people with a history of suicide attempts than those without. From the viewpoint of Klonsky and May (2014)’s ideation to action framework, these results suggest that those who have a greater severity of ASAD symptoms, among those who have had suicidal ideation, may be more likely to engage in suicidal actions than those who have not. However, no significant group difference was found in ASAD symptoms between single attempters and multiple attempters. These findings suggest that higher levels of ASAD symptoms could differentiate people who only think about from those who attempt suicide, although we cannot predict who is going to attempt multiple times using solely levels of ASAD symptoms. This may be due to the fact that the ASADI-L measures lifetime worst-point symptoms rather than current symptoms or trait-like characteristics of multiple attempters. Investigations using the current version of the ASADI (ASADI-C), which assesses ASAD symptoms for the past week, or a prospective design will be able to explore clinical symptomatology of single versus multiple attempters. Further research to explore a relevant cut-off score of the ASADI-L to classify the suicide risk level is warranted.

Tucker et al. (2016) defined acute suicidal crisis as experiencing a drastic increase in suicidal intention “over the course of hours or days,” proposing that for those who respond “No” to the ASADI-L item 2a (“In your lifetime, have you ever experienced a drastic increase in your intent to kill yourself?”) or 2b (“If yes, did this drastic increase in intent to kill yourself occur over the course of hours or days, not over the course of weeks or months?”), the remaining questions should be coded as “0,” discontinuing the questionnaire. However, this study continued to collect subsequent responses if a participant responded “Yes” to the ASADI-L item 2a, regardless of the response to 2b. The purposes of this approach were to avoid possibly excluding people who experienced a drastic increase in suicidal ideation for a period longer than several hours or days from an important risk group and to examine whether there are differences in the severity of ASAD symptoms. Thus, this study compared the acute suicidal intention group, in which there was a drastic increase in suicidal intention within several hours or days, and the non-acute suicidal intention group, in which the drastic increase in suicidal intention occurred over a longer period.

According to the study results, the acute suicidal intention group showed a significantly higher level of ASAD symptom severity than the non-acute suicidal intention group, in addition to higher levels of C-SSRS suicidal ideation severity, depression, anxiety, and self-injurious behavior. For each item of the ASADI-L, significantly higher severity levels were found in the acute suicidal intention group than the non-acute suicidal intention group for all ASAD symptoms, except for hopelessness that social alienation is unlikely to disappear and some symptoms of overarousal (agitation, irritability, insomnia). Overall, these results suggest that the acute and non-acute suicidal intention groups differ in terms of severity of ASAD symptoms and clinical symptoms of depression or anxiety. Compared to the non-acute suicidal intention group, the acute group reported more symptoms related to social/self-isolation, consistent with interpersonal-psychological theory.

However, it is noteworthy that there was no significant difference between the acute and non-acute suicidal intention groups in history of suicide attempts. This means that these two groups did not differ in engaging in a suicide attempt upon a drastic increase of suicidal intention regardless of the duration of the suicide intention. This affirms the need for not excluding people with a drastic suicidal intention for a longer period in assessing ASAD symptoms using the ASADI-L. That is, if a participant responded “Yes” to Question 2a of the ASADI-L, regardless of the response to Question 2b, the assessment should not be discontinued. Further investigation is needed to compare clinical characteristics and future suicide risk of those two subgroups.

While previous validation studies of the ASADI-L were conducted using a sample of college students, psychiatric outpatients, or inpatients in the United States (Stanley et al., 2016; Tucker et al., 2016; Rogers et al., 2017a), the current study provided solid evidence for the one factor model of ASAD symptoms among community adults in a non-western country. However, the question that ASAD symptoms would predict near-term suicide attempt in the future or suicide remains unanswered. In a related vein, it is also unknown whether ASAD symptoms would have better predictive validity for future suicide attempt than current suicidal ideation severity and past suicidal behavior, which are widely used indexes of suicide risk. Due to the ASAD instruction, only individuals who have experienced suicidal ideation and a drastic increase in suicidal intention responded to subsequent items on suicide crisis symptoms. Yet, whether it is a relevant screening criterion in community adults needs verification. For example, the Suicidal Crisis Inventory-2 (SCI-2; Bloch-Elkouby et al., 2021), a measure of SCS (Galynker et al., 2017) does not include items asking suicidal ideation or intent and thus all participants can respond to suicide crisis symptom items. Which method is more efficient in identifying people at risk in the community needs further investigation.

From a clinical point of view, the ASADI-L provides more clinically useful information than the conventional suicidal behavior measures, such as the C-SSRS, a widely used measure of suicidal ideation severity and suicide attempt history. In this respect, when encountering a person experiencing a high level of suicidal ideation and a drastic increase in suicidal intention in a clinical setting, the ASADI-L will be able to provide insights on psychosocial intervention by addressing the social/self-alienation, overarousal symptoms, and hopelessness. Thus, clinicians can provide personalized treatment plan to reduce suicide risk.

Limitations of this study are described as follows. First, the ASADI-L measures lifetime worst-point ASAD symptoms, which may or may not have aligned with the timing of past suicide attempts. We cannot definitively say that ASAD symptoms preceded past suicide attempts either. Thus, there is a need for further studies that verify the predictive power of ASAD symptoms for future suicidal behavior using longitudinal data. Second, most of the participants in this study were women. This may be due to the sampling strategy of this study (i.e., web-based survey without using a stratified sampling method). Thus, it is limited to generalize the findings to men. Further investigation is needed to verify the validity of the scale is equally applicable to all genders. Third, because this study was conducted with community adults, it is necessary to verify whether similar results are obtained in clinical samples receiving treatment due to suicide crises. Overall, the lack of external validity is a main limitation of this study and warrants further research.

Despite these limitations, this study has significance in that the factor structure and validity of the ASADI-L are consistent with Tucker et al. (2016)’s study using an American sample, supporting the international use of the measure. Furthermore, this study expanded Tucker et al.’s study by examining ASAD symptoms and clinical characteristics of the acute and non-acute suicidal intention group. Recently, DSM-5-TR (American Psychiatric Association, 2022) assigned an independent diagnostic code to suicidal behavior, despite the lack of research in the diagnostic criteria for suicidal crisis. The current study could provide preliminary evidence for establishing the criteria and provide useful information regarding appropriate clinical interventions for those at acute risk of suicide.

## Data availability statement

The raw data supporting the conclusions of this article will be made available by the authors, without undue reservation.

## Ethics statement

The studies involving human participants were reviewed and approved by Chungbuk National University. The patients/participants provided their written informed consent to participate in this study.

## Author contributions

YO and SY designed the study. SL and YO collected and analyzed the data. YO drafted the manuscript. SY and MR provided critical revisions. All authors contributed to the article and approved the submitted version.

## Funding

This work was supported by the Ministry of Education of the Republic of Korea and the National Research Foundation of Korea (NRF-2020S1A5A2A03044181).

## Conflict of interest

The authors declare that the research was conducted in the absence of any commercial or financial relationships that could be construed as a potential conflict of interest.

## Publisher’s note

All claims expressed in this article are solely those of the authors and do not necessarily represent those of their affiliated organizations, or those of the publisher, the editors and the reviewers. Any product that may be evaluated in this article, or claim that may be made by its manufacturer, is not guaranteed or endorsed by the publisher.
