# Supplementary material for: Assessment of acute and non-acute suicide crisis symptoms: Validation of the Korean version of the acute suicidal affective disturbance inventory
Source: Front Psychol. 2022 Nov 18;13:1034130. doi: 10.3389/fpsyg.2022.1034130 (PMC9716654; doi:10.3389/fpsyg.2022.1034130)
Supplement: Supplementary file 1 [file Data_Sheet_1.docx]

Supplementary Material

**급성 자살 정동 장애 척도**

**Acute Suicidal Affective Disturbance Inventory–Lifetime (ASADI-L)**

각 문항을 잘 읽고 당신의 일생동안의 경험을 바탕으로 응답해 주십시오.

1. 지금까지 살면서, 자살을 생각해 본 적이 있습니까?

예___ 아니오____

| ‘아니오’라면 응답을 마치십시오. ‘예’라면 2a 문항에 응답하세요. |
| --- |

다음 질문은 자살의도에 관한 것입니다. 자살의도란 실제로 자살행동에 관여하고 죽고 싶은 욕구에 따라 행동할 가능성을 말합니다.

2a. 지금까지 살면서, 자살하고 싶은 의도가 급격하게 높아졌던 적이 있습니까?

예___ 아니오___

| ‘아니오’라면 응답을 마치십시오. ‘예’라면 2b 문항에 응답하세요. |
| --- |

2b. 만약 위의 질문에 ‘예’라고 답하셨다면, 자살의도가 급격하게 높아졌던 경험이 몇 시간 또는 며칠 사이에 발생한 것이었습니까? (몇 주 또는 몇 달의 기간동안 발생한 것이 아님)

예___ 아니오___

2c. 자살의도가 가장 급격하게 높아졌던 위의 경험 당시 자살의도가 어느 정도였는지 그 범위에 해당하는 숫자 두 개를 아래의 빈 칸에 각각 적어주십시오.

| 0 | 1 | 2 | 3 | 4 | 5 | 6 | 7 | 8 | 9 | 10 |
| --- | --- | --- | --- | --- | --- | --- | --- | --- | --- | --- |
| 의도  없음 | 매우  낮은  의도 |  |  |  | 중간  정도의  의도 |  |  |  |  | 매우  높은  의도 |

범위:___(가장 낮은 의도)에서____(가장 높은 의도)

당신은 지금까지 살면서, 몇 시간 또는 며칠 이내에 자살의도가 급격하게 높아진 경험이 있다고 답했습니다. 만약 이러한 경험이 한 번 이상 존재한다면, 가장 심각했던 경험을 떠올려 보시고 오직 그 경험만을 바탕으로 아래의 질문에 답해주십시오.

자살의도가 급격히 높아졌을 때 중 가장 심각했을 때가 언제인지 연도와 월을 적어주세요:

___년___월

3. 자살의도가 급격히 높아졌을 때 중 가장 심각했을 때, 자살 생각이 없어지거나 변하지 않을 것 같은 느낌을 얼마나 받았습니까?

| 0 | 1 | 2 | 3 | 4 | 5 | 6 | 7 | 8 | 9 | 10 |
| --- | --- | --- | --- | --- | --- | --- | --- | --- | --- | --- |
| 전혀 아님 |  |  |  |  | 중간  정도로 |  |  |  |  | 매우  심하게 |

4a. 자살의도가 급격히 높아졌을 때 중 가장 심각했을 때, 다른 사람들과 단절된 느낌을 받았습니까?

예___ 아니오___

4b. 자살의도가 급격히 높아졌을 때 중 가장 심각했을 때, 다른 사람들과 단절된 느낌을 얼마나 받았습니까?

| 0 | 1 | 2 | 3 | 4 | 5 | 6 | 7 | 8 | 9 | 10 |
| --- | --- | --- | --- | --- | --- | --- | --- | --- | --- | --- |
| 전혀 아님 |  |  |  |  | 중간  정도로 |  |  |  |  | 매우  심하게 |

4c. 자살의도가 급격히 높아졌을 때 중 가장 심각했을 때, 다른 사람들과 단절된 느낌이 없어지거나 변하지 않을 것 같은 느낌을 얼마나 받았습니까?

| 0 | 1 | 2 | 3 | 4 | 5 | 6 | 7 | 8 | 9 | 10 |
| --- | --- | --- | --- | --- | --- | --- | --- | --- | --- | --- |
| 전혀 아님 |  |  |  |  | 중간  정도로 |  |  |  |  | 매우  심하게 |

5a. 자살의도가 급격히 높아졌을 때 중 가장 심각했을 때, 다른 사람과 함께 있는 것이 극도로 싫거나 혐오스러웠습니까?

예___ 아니오___

5b. 자살의도가 급격히 높아졌을 때 중 가장 심각했을 때, 다른 사람과 함께 있는 것이 얼마나 싫거나 혐오스러웠습니까?

| 0 | 1 | 2 | 3 | 4 | 5 | 6 | 7 | 8 | 9 | 10 |
| --- | --- | --- | --- | --- | --- | --- | --- | --- | --- | --- |
| 전혀 아님 |  |  |  |  | 중간  정도로 |  |  |  |  | 매우  심하게 |

5c. 자살의도가 급격히 높아졌을 때 중 가장 심각했을 때, 다른 사람과 함께 있는 것이 극도로 싫거나 혐오스러운 감정이 없어지거나 변하지 않을 것 같은 느낌을 얼마나 받았습니까?

| 0 | 1 | 2 | 3 | 4 | 5 | 6 | 7 | 8 | 9 | 10 |
| --- | --- | --- | --- | --- | --- | --- | --- | --- | --- | --- |
| 전혀 아님 |  |  |  |  | 중간  정도로 |  |  |  |  | 매우  심하게 |

6a. 자살의도가 급격히 높아졌을 때 중 가장 심각했을 때, 당신이 없는 것이 주변 사람들에게 더 나을 것 같다는 느낌을 강하게 받았습니까?

예___ 아니오___

6b. 자살의도가 급격히 높아졌을 때 중 가장 심각했을 때, 당신이 없는 것이 주변 사람들에게 더 나을 것 같다는 느낌을 얼마나 받았습니까?

| 0 | 1 | 2 | 3 | 4 | 5 | 6 | 7 | 8 | 9 | 10 |
| --- | --- | --- | --- | --- | --- | --- | --- | --- | --- | --- |
| 전혀 아님 |  |  |  |  | 중간  정도로 |  |  |  |  | 매우  심하게 |

6c. 자살의도가 급격히 높아졌을 때 중 가장 심각했을 때, 당신이 없는 것이 주변 사람들에게 더 나을 것 같다는 느낌이 없어지거나 변하지 않을 것 같은 느낌을 얼마나 받았습니까?

| 0 | 1 | 2 | 3 | 4 | 5 | 6 | 7 | 8 | 9 | 10 |
| --- | --- | --- | --- | --- | --- | --- | --- | --- | --- | --- |
| 전혀 아님 |  |  |  |  | 중간  정도로 |  |  |  |  | 매우  심하게 |

7a. 자살의도가 급격히 높아졌을 때 중 가장 심각했을 때, 나 자신에 대한 증오심이나 혐오감을 강하게 느꼈습니까?

예___ 아니오___

7b. 자살의도가 급격히 높아졌을 때 중 가장 심각했을 때, 나 자신에 대한 증오심이나 혐오감을 얼마나 강하게 느꼈습니까?

| 0 | 1 | 2 | 3 | 4 | 5 | 6 | 7 | 8 | 9 | 10 |
| --- | --- | --- | --- | --- | --- | --- | --- | --- | --- | --- |
| 전혀 아님 |  |  |  |  | 중간  정도로 |  |  |  |  | 매우  심하게 |

7c. 자살의도가 급격히 높아졌을 때 중 가장 심각했을 때, 나 자신에 대한 증오심이나 혐오감이 끝나거나 변하지 않을 것 같은 느낌을 얼마나 받았습니까?

| 0 | 1 | 2 | 3 | 4 | 5 | 6 | 7 | 8 | 9 | 10 |
| --- | --- | --- | --- | --- | --- | --- | --- | --- | --- | --- |
| 전혀 아님 |  |  |  |  | 중간  정도로 |  |  |  |  | 매우  심하게 |

아래 숫자를 사용하여 가장 심각하게 자살의도가 급격히 높아졌던 당시에, 다음을 얼마나 경험했는지 답해주십시오(8-11a):

8. 초조감, 불편감, 안절부절못함

| 0 | 1 | 2 | 3 | 4 | 5 | 6 | 7 | 8 | 9 | 10 |
| --- | --- | --- | --- | --- | --- | --- | --- | --- | --- | --- |
| 전혀 아님 |  |  |  |  | 중간  정도로 |  |  |  |  | 매우  심하게 |

9. 매우 짜증남

| 0 | 1 | 2 | 3 | 4 | 5 | 6 | 7 | 8 | 9 | 10 |
| --- | --- | --- | --- | --- | --- | --- | --- | --- | --- | --- |
| 전혀 아님 |  |  |  |  | 중간  정도로 |  |  |  |  | 매우  심하게 |

10. 잠에 들기 어렵거나 수면을 유지하기 어렵거나 너무 일찍 잠에서 깸

| 0 | 1 | 2 | 3 | 4 | 5 | 6 | 7 | 8 | 9 | 10 |
| --- | --- | --- | --- | --- | --- | --- | --- | --- | --- | --- |
| 전혀 아님 |  |  |  |  | 중간  정도로 |  |  |  |  | 매우  심하게 |

11a. 악몽을 꿈

| 0 | 1 | 2 | 3 | 4 | 5 | 6 | 7 | 8 | 9 | 10 |
| --- | --- | --- | --- | --- | --- | --- | --- | --- | --- | --- |
| 전혀 아님 |  |  |  |  | 중간  정도로 |  |  |  |  | 매우  심하게 |

11b. 자살의도가 급격히 높아졌을 때 중 가장 심각했을 때, 악몽을 몇 번이나 꾸었습니까?

_____번

12. 자살의도가 급격히 높아졌을 때 중 가장 심각했을 때, 당신이 경험했던 문제들(문항 1-11) 중 약물 또는 음주로 인해 발생한 것이 있었습니까?

예___ 아니오___

13. 자살의도가 급격히 높아졌을 때 중 가장 심각했을 때, 당신이 경험했던 문제들(문항 1-11) 중 진행 중인 우울증이 갑작스럽게 악화되어 발생한 것이 있었습니까?

예___ 아니오___

Scoring: 2c*10 + (4b*4c+5b*5c+6b*6c)/3 + 7b*7c + [8+9+10+11a]/4*10
